# Long noncoding RNA PVT1 promotes breast cancer proliferation and metastasis by binding miR-128-3p and UPF1

**DOI:** 10.1186/s13058-021-01491-y

**Published:** 2021-12-18

**Authors:** Shuiyi Liu, Weiqun Chen, Hui Hu, Tianzhu Zhang, Tangwei Wu, Xiaoyi Li, Yong Li, Qinzhi Kong, Hongda Lu, Zhongxin Lu

**Affiliations:** 1 Department of Medical Laboratory, The Central Hospital of Wuhan, Tongji Medical College, Huazhong University of Science and Technology, 26 Shengli St., Jiangan District, Wuhan, 430014 China; 2 Cancer Research Institute of Wuhan, The Central Hospital of Wuhan, Tongji Medical College, Huazhong University of Science and Technology, Wuhan, 430014 China; 3 Key Laboratory for Molecular Diagnosis of Hubei Province, The Central Hospital of Wuhan, Tongji Medical College, Huazhong University of Science and Technology, Wuhan, 430014 China; 4 School of Laboratory Medicine, Hubei University of Chinese Medicine, Wuhan, 430065 China; 5 Department of Medicine, Dan L Duncan Comprehensive Cancer Center, Baylor College of Medicine, Houston, TX 77030 USA; 6 Department of Oncology, The Central Hospital of Wuhan, Tongji Medical College, Huazhong University of Science and Technology, Wuhan, 430014 China

**Keywords:** Plasmacytoma variant translocation 1, Breast cancer, Epithelial–mesenchymal transition, miR-128-3p, Up-frameshift protein 1

## Abstract

**Background:**

Mounting evidence supports that long noncoding RNAs (lncRNAs) have critical roles during cancer initiation and progression. In this study, we report that the plasmacytoma variant translocation 1 (PVT1) lncRNA is involved in breast cancer progression.

**Methods:**

qRT-PCR and western blot were performed to detect the gene and protein expression. Colony formation would healing and transwell assays were used to detect cell function. Dual-luciferase reporter assay and RNA pull-down experiments were used to examine the mechanisms interaction between molecules. Orthotopic mouse models were established to evaluate the influence of PVT1 on tumor growth and metastasis in vivo.

**Results:**

PVT1 is significant upregulated in breast cancer patients’ plasma and cell lines. PVT1 promotes breast cancer cell proliferation and metastasis both in vitro and in vivo. Mechanistically, PVT1 upregulates FOXQ1 via miR-128-3p and promotes epithelial–mesenchymal transition. In addition, PVT1 binds to the UPF1 protein, thereby inducing epithelial–mesenchymal transition, proliferation and metastasis in breast cancer cells.

**Conclusion:**

PVT1 may act as an oncogene in breast cancer through binding miR-128-3p and UPF1 and represents a potential target for BC therapeutic development.

**Supplementary Information:**

The online version contains supplementary material available at 10.1186/s13058-021-01491-y.

## Background

In 2019, breast cancer (BC) is the most prevalent malignancy among women worldwide and is the second leading cause of cancer-associated death in women after lung and bronchus cancer, according to the American Cancer Society [1]. In recent decades, great improvements have been made in diagnosis, surgery, chemotherapy, and molecular targeted treatment on BC. Yet, the prognosis for Stage IV BC is far from satisfactory because of its heterogeneity and complexity. Therefore, molecular pathogenesis of BC should be further investigated to identify better targets for therapeutic development.

Long noncoding RNAs (lncRNAs), previously regarded as transcriptional noise, have gradually become a focus of research in various diseases, especially in cancer [2, 3]. LncRNAs are endogenous cellular molecules with more than 200nt. Lacking an open reading frame, lncRNAs have limited protein-coding capacity [4]. However, accumulating evidence has demonstrated that the abnormal lncRNAs expression is involved in a variety of biological and pathological processes, such as cell proliferation, cell apoptosis, cell invasion, cell metastasis, stem cell renewal, drug resistance, and so on [5, 6].

The plasmacytoma variant translocation 1 (PVT1) is an oncogenic lncRNA, located at the 8q24 region, was discovered in murine plasmacytoma in 1985 [7]. PVT1 is upregulated in numerous cancers, functioning as an oncogene in malignant progression [8, 9]. PVT1 directly interacts with p-STAT3 to activate the STAT3 signaling pathway, thereby promoting angiogenesis in gastric cancer [10]. Recent research has revealed that PVT1 is up regulated in nasopharyngeal carcinoma tissues and its overexpression predicts a poor prognosis for NPC patients. Loss-of-function experiments support that PVT1 regulates cell apoptosis by influencing the DNA damage repair pathway after radiation, suggesting that targeting PVT1 may be a potential strategy for NPC therapy [11]. However, more data are required to elucidate the functions of PVT1 in tumor progression.

In our study, we report that PVT1 is highly expressed both in plasma from BC patients and BC cells. In addition, we show that PVT1 promotes BC proliferation and metastasis both in vitro and vivo. Mechanistically, PVT1 directly binds UPF1 and competitively recruits miR-128-3p to upregulate FOXQ1, thereby promoting BC progression.

## Materials and methods

### Clinical specimens

In this study, blood samples were collected from female patients at the Central Hospital of Wuhan, including 80 BC patients, 70 healthy volunteers, and 70 fibroma patients; for 35 BC patients, paired blood samples (preoperative and postoperative blood in one month after surgery) were collected. None of the patients had received any clinical treatment before sample collection and corresponding tumor specimens were confirmed by histopathological examination. No diseases or injuries were found in the healthy controls. Peripheral blood samples were collected in EDTA-containing tubes and were processed within 4 h by centrifugation at 1000 g for 15 min at 4 °C. The supernatant was then transferred into RNAse-free tubes and stored at − 80 °C. This study was approved by the Medical Ethics Committee of The Central Hospital of Wuhan and all human subject research was performed in accordance with institutional, national, and Declaration of Helsinki requirements.

### Cell culture

The human BC cell lines (Hs578t, MCF-7, MDA-MB-231, T47D, Bt549, HCC1806), the noncancerous breast epithelial HBL-100 cells, and the human embryonic kidney (HEK) 293 T cells were purchased from the American Type Culture Collection (ATCC). Cell culture was performed following the recommendations of ATCC. We confirm the authentication of all cell lines used—the full policy and requirements are available in the instructions to authors.

### Plasmids construction and cell transfection

The pSIF–GFP–miR-128-3p precursor plasmid and the precursor control were gifts from Dr. Yong Li in Baylor College of Medicine. miR-128-3p inhibitors and miRNA controls were obtained from GenePharma Technology (Shanghai, China). Small interfering RNAs (siRNAs) targeting PVT1 and a negative control were also obtained from GenePharma Technology (Shanghai, China). Three shRNAs targeting human UPF1 and the shRNA negative control sequence were as follows: shUPF1#1, sense: 5’-GATCCGAGCCACATTGTAAATCATTTCAAGAGAATGATTTA CAATGTGGCTTTTTTTG-3, antisense: 5’-AATTCAAAAAAAGCCACATTGTAAATC ATTCTCTTGAAATGATTTACAATGTGGCTCG-3’; shUPF1#2, sense: 5’-GATCCG GCGAGAAGGACTTCATCATTCAAGAGATGATGAAGTCCTTCTCGCTTTTTTG-3’, antisense: 5’-AATTCAAAAAAGCGAGAAGGACTTCATCATCTCTTGAATGATGAA GTCCTTCTCGCCG-3’; shUPF1#3, sense: 5’-GATCCGGCAGCCACATTGTAAAT.

CATCAAGAGTGATTTACAATGTGGCTGCTTTTTTG-3’, antisense: 5’-AATTCA AAAAAGCAGCCACATTGTAAATCACTCTTGATGATTTACAATGTGGCTGCCG-3’. The shRNA constructs were cloned into Bam HI and Eco RI sites in an RNAi-ready pSIREN-RetroQ vector (Clontech, USA). All DNA constructs were confirmed by Sanger sequencing. Transfections were performed using Lipofectamine® LTX and Plus reagent (Invitrogen, USA) according to the manufacturer’s instructions.

### Colony formation assay

After transfection, Hs578t or MCF-7 cells were seeded into 12-well plates at 500 cells/well and cultured in DMEM with 10% FBS at 37 °C for 2 weeks. Plates were washed twice with PBS, colonies were then fixed with 4% paraformaldehyde for 20 min and stained with 0.1% crystal violet for 30 min. More than 50 cells were counted as a colony.

### RNA immunoprecipitation (RIP) assay

RIP was performed using a EZ-Magna RIP kit (Millipore, USA) according to the manufacturer’s instructions. In brief, Hs578t or MCF-7 cells were lysed in the complete RIP lysis buffer and then the lysates were incubated with beads-antibody in RIP buffer. Rabbit anti-UPF1 antibody and rabbit IgG control (Millipore, USA) were used. Finally, the co-precipitated RNAs were extracted and detected by qRT-PCR.

### Wound healing assay

Hs578t or MCF-7 cells were seeded in 6-well plates. A wound was made by a sterile 200 μl pipette tip 24 h post transfection. The images (0 h) were taken using a microscope. After incubation at 37 ℃ for 24 h, cells were washed twice with PBS and the wound healing images (24 h) were photographed again.

### Transwell assay

To investigate the capacities of cell invasion, transwell chamber (Corning, USA) was coated with Matrigel (BD Biosciences, USA). Hs578t or MCF-7 cells (5 × 10^4^) were starved in 200 μL serum-free medium 24 h post transfection and then seeded into the top chamber of each insert (8 μm, Corning, USA). The lower chambers were filled with 600 μL of complete medium. After cultured at 37 ℃ for 24 h, cells migrated to the lower chambers were fixed, stained, and counted.

### RNA isolation and qRT-PCR

RNA was extracted using TRIzol reagent (Invitrogen, USA). cDNA was quantified by SYBR green (Applied BioSystems, USA) on the StepOne System (Bio-Rad, USA). U6 was used as the endogenous control for miR-128-3p and GAPDH for PVT1. Each reaction was performed in triplicates and relative expression of target genes was calculated by the 2^–△△Ct^ method for gene expression in BC cells and the 2^–△Ct^ method for that in BC plasma. The following primers were used: PVT1 sense (5’-TGAGAACTGTCCTTA CGTGACC-3’) and antisense (5’-AGAGCACCAAGACTGGCTCT-3’); GAPDH sense (5’-GGGAGCCAAAAGGGTCAT-3’) and antisense (5’-GAGTCCTTCC ACGATACCAA-3’).

### Western blot assay

Western blot analyses were conducted according to the method described previously [12]. The primary antibodies were: rabbit monoclonal anti-E-cadherin (#3195, 1:1000, Cell Signaling Technology), rabbit monoclonal anti-Vimentin (#5741, 1:1000, Cell Signaling Technology), rabbit polyclonal anti-UPF1 (23379-1-AP, 1:1000, ProteinTech), rabbit polyclonal anti-FOXQ1 (23718-1-AP, 1:1000, ProteinTech), mouse monoclonal anti-β-actin (A5316, 1:5000, Sigma-Aldrich), and mouse monoclonal anti-PCNA (sc-25280, 1:1000, Santa Cruz). The blot signal was detected using ECL (GE Healthcare, UK).

### Dual-luciferase reporter assay

For the PVT1 luciferase reporter assay, pRL-TK-PVT1 or pRL-TK-PVT1 mutant vectors and pGL3 control vector (Promega) were co-transfected into HEK293T along with pSIF-GFP-miR-128-3p precursor plasmid using Lipofectamine® LTX and the Plus reagent (Invitrogen).

For miR-128-3p target gene FOXQ1 luciferase reporter assay, pRL-TK-FOXQ1-3΄UTR or pRL-TK-FOXQ1-3΄UTR mutant vectors and pGL3 control vector (Promega) were co-transfected into cells along with pSIF-GFP-miR-128-3p precursor plasmid using Lipofectamine® LTX and the Plus reagent (Invitrogen). Luciferase activity was detected 48 h after transfection using the Dual-Glo luciferase reporter assay system (Promega) according to the manufacturer’s instructions. Experiments were repeated at least three times.

### In vivo experiments

Female athymic 4-week-old BALB/c nude mice were purchased from HFK Bio-Technology. Co., Ltd (Beijing, China). Hs578t (5 × 10^6^ cells) were injected into the mammary fat pad. Tumor volumes were analyzed using the formula *V* = length × width^2^/2. When the tumor volumes reached 60 mm^3^, the mice were randomly divided into a control group (*n* = 6) and a si-PVT1 group (*n* = 6). si-PVT1 or the siRNA control (20 μl, 5 nM)) was directly injected into the implanted tumor twice per week. After 3 weeks, all the nude mice were anesthetized by 1% pentobarbital sodium (50 mg/kg) and euthanized through the cervical vertebra acetabular method. The tumors and major organs were fixed in 10%-buffered formalin and embedded in paraffin for immunohistochemistry or hematoxylin & eosin (H&E) staining.

### Statistical analysis

All statistical analyses were carried out using Graphpad Prism 5.0 software. Student’s t-test or one-way analysis of variance (ANOVA) were performed for comparisons between groups. *P* value < 0.05 indicates statistical significance. *P < 0.05, **P < 0.01, ***P < 0.001.

## Results

### PVT1 is significantly up-regulated in BC cell lines and plasma

**Fig. 1:**
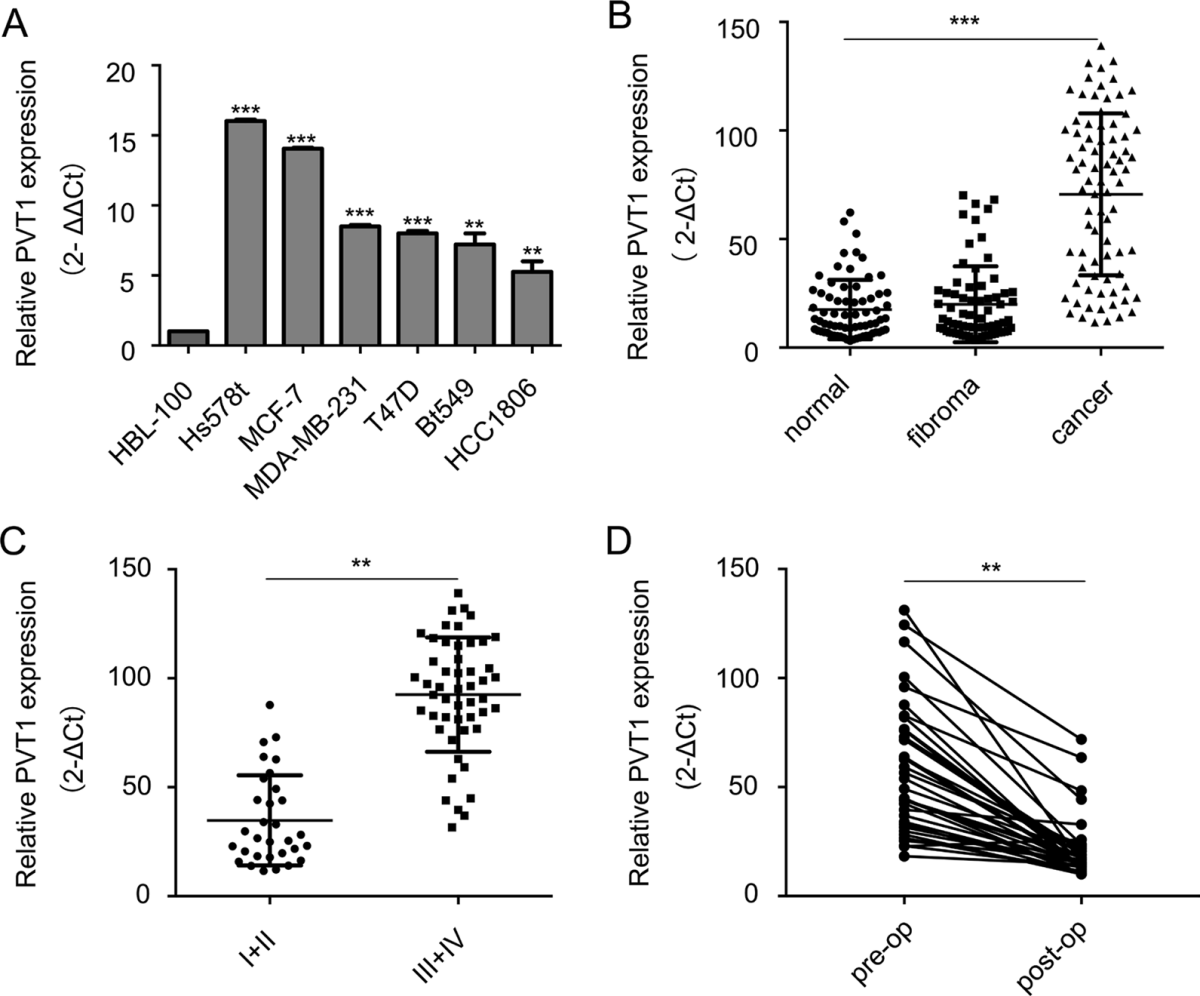
The expression of PVT1 in BC cell lines and patients’ plasma. **A** The expression levels of PVT1 in BC cell lines and the breast epithelial cell line HBL-100. **B** The plasma levels of PVT1 in 70 healthy controls, 70 fibroma patients and 80 BC patients. **C** The plasma levels of PVT1 of BC patients at different stages. **D** The plasma levels of PVT1 in 35 BC patients with paired preoperative and postoperative specimens. Results were presented as the mean ± SD. **p < 0.01, ***P < 0.001

To explore the expression profile of PVT1 in BC, we first detected PVT1 expression levels in BC cell lines. PVT1 expressions were remarkably higher in BC cells than that in the immortalized mammary epithelial cell line HBL-100 cells (Fig. 1A). Moreover, we tested PVT1 expression levels in plasma samples from 80 BC patients, 70 fibroma patients, and 70 healthy controls. PVT1 levels were significantly increased in BC samples compared with either healthy controls or breast fibroma patients (Fig. 1B). Breast fibromatosis is the most common histologically benign lesion and rarely undergo malignant transformation. The clinical characteristics of the BC patients are listed in Table 1. To further explore the significance of PVT1 expression in BC, we analyzed PVT1 expression in relationship to tumor stages and found that the plasma levels of PVT1 were significantly higher in patients with stage III and IV than those with stage I and II (Fig. 1C). And we also find the expression of PVT1 has no difference among the subtypes (see Additional file 1: Table). In addition, we analyzed 35 paired preoperative and postoperative plasma samples from BC patients, PVT1 expressions were remarkably reduced in the postoperative samples (Fig. 1D). These results suggested that PVT1 is a candidate predictor for BC diagnosis and staging assessment. Because the PVT1 expression was the highest in cell line Hs578t and MCF-7, we chose these two lines for further study.

**Table 1:** Association between PVT1 expression and the clinicopathological characteristics of breast cancer patients (all female)

Characteristics	Case (*n* = 80)	Expression of PVT1		*P* value
		Low (*n* = 40)	High (*n* = 40)	
*Age*				
≤ 50	35	16	19	0.499
> 50	45	24	21	
*Tumor size*				
≤ 3 cm	33	11	22	0.012
> 3 cm	47	29	18	
*TNM stage*				
I–II	31	21	10	0.012
III–IV	49	19	30	
*LN metastasis*				
Yes	42	15	27	0.007
No	38	25	13	
*ER status*				
Positive	30	13	17	0.356
Negative	50	27	23	
*PR status*				
Positive	37	16	21	0.361
Negative	43	23	20	
*HER2 status*				
Positive	36	16	20	0.369
Negative	44	24	20	

### PVT1 promotes BC cell proliferation, migration and invasion in vitro

**Fig. 2:**
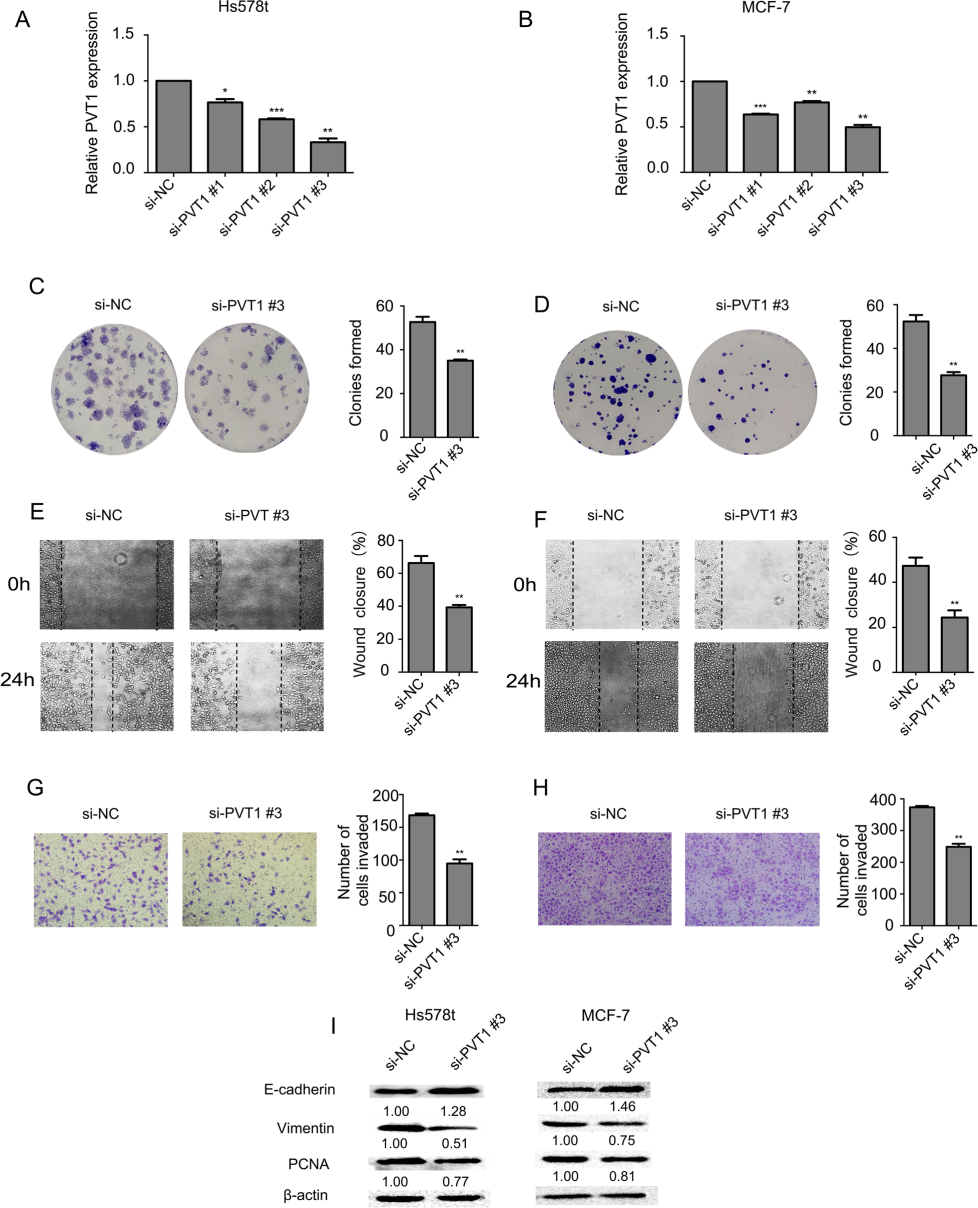
Inhibition of PVT1 on cell proliferation, migration and invasion of Hs578t and MCF-7 cells. **A** and **B**: si-PVT1 knockdown efficiency in Hs578t and MCF-7 cells. **C** and **D**: Colony formation of cells transfected with si-PVT1 or si-NC. **E** and **F**: Wound healing assays for cell migration. **G** and **H**: Transwell assays for cell invasion. **I:** Western blot analyses of proteins that are involved in cell proliferation and metastasis. Results were presented as the mean ± SD. *p < 0.05, **p < 0.01, ***P < 0.001

To investigate the function consequences of PVT1 overexpression on BC, we introduced three specific si-PVT1s into BC cells. si-PVT1 #3 was the most efficient in knocking down PVT1 expression (Fig. 2A, see Additional file 1: Figure A). The colony formation assay showed that knockdown of PVT1 significantly inhibited Hs578t and MCF-7 cell proliferation (Fig. 2B, C). Additionally, the wound healing assay showed that cells transfected with si-PVT1 underwent a slower closing of scratch wound than the control (Fig. 2D, E). Meanwhile, the transwell assay showed that knockdown of PVT1 dramatically decreased Hs578t and MCF-7 cell invasion (Fig. 2F, G). These findings indicate that PVT1 promotes BC cell proliferation, migration and invasion in vitro. We examined protein markers for cancer proliferation, migration and invasion in Hs578t and MCF-7 cells. As shown in Fig. 2H, following the treatment of the si-PVT1, the level of the epithelial marker E-cadherin was upregulated, while that of the mesenchymal marker Vimentin and proliferation-related protein PCNA was downregulated in Hs578t and MCF-7 cells. These results indicate that inhibition of PVT1 suppresses cell proliferation, migration, invasion and EMT in BC.

### PVT1 promotes BC growth and metastasis in vivo

**Fig. 3:**
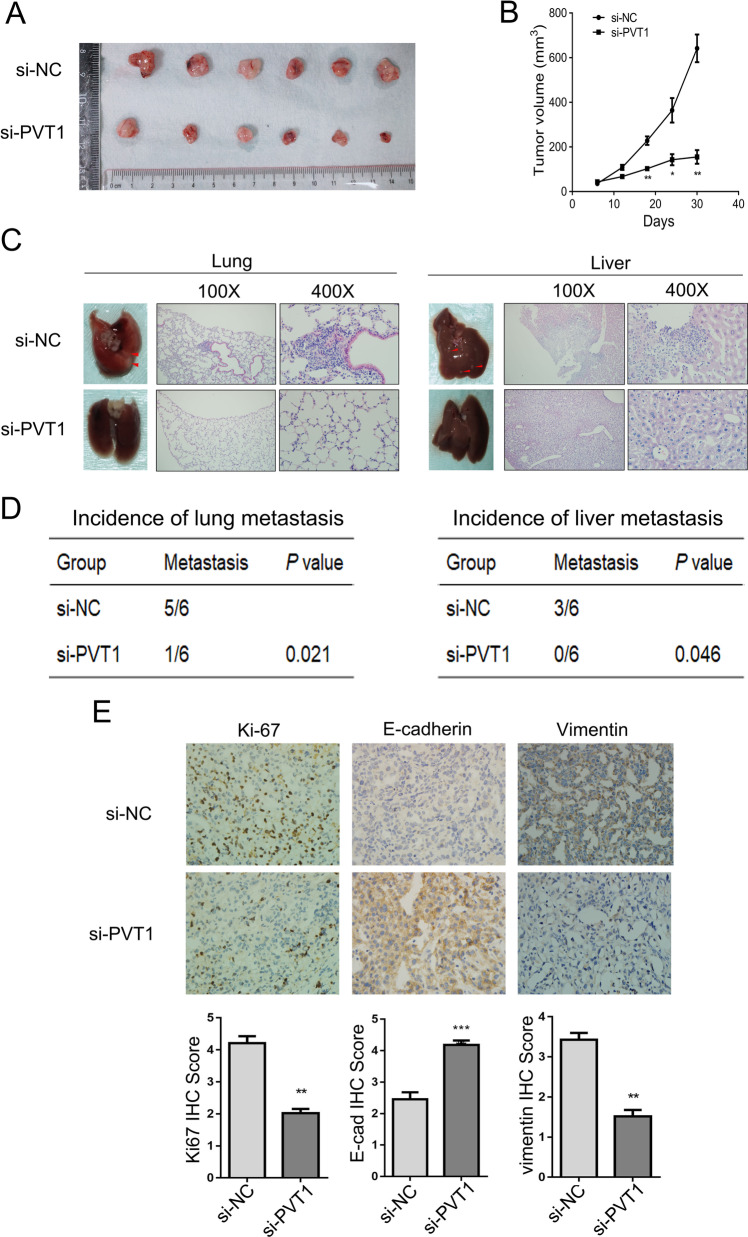
Downregulation of PVT1 reduces tumor growth and metastasis in vivo. **A** Tumor tissues from nude mice treated with si-NC and si-PVT1 (*n* = 6 for each group). **B** Tumor volumes in two groups were evaluated. **C** Lung and liver tissues were obtained, and the metastatic cells were visualized. **D** The incidence of lung and liver metastasis in mice was shown in the table. **E** IHC analyses of Ki-67, E-cadherin, and vimentin in xenografted tumors

To further evaluate the effects of PVT1 in vivo, we injected the si-PVT1 or a negative control intratumorally, when the tumors xenografted with Hs578t cells reached an average of 60 mm^3^ in immunodeficient mice. We found that the volumes of tumors in mice treated with the si-PVT1 were markedly smaller than those in mice injected with the control (Fig. 3A, B). Lung and liver samples were obtained to evaluate tumor metastasis, and the number of metastatic cells in liver and lung were significantly reduced by si-PVT1 (Fig. 3C, D). In addition, immunohistochemistry results (Fig. 3E) showed that the expression levels of Ki-67 and vimentin were much lower and that of E-cadherin was higher in tumors treated with si-PVT1 than that with the control. These results implicate that downregulation of PVT1 significantly reduces tumor growth and metastasis in vivo.

### PVT1 function as a competing endogenous RNA and regulates FOXQ1 expression by competitively binding miR-128-3p

**Fig. 4:**
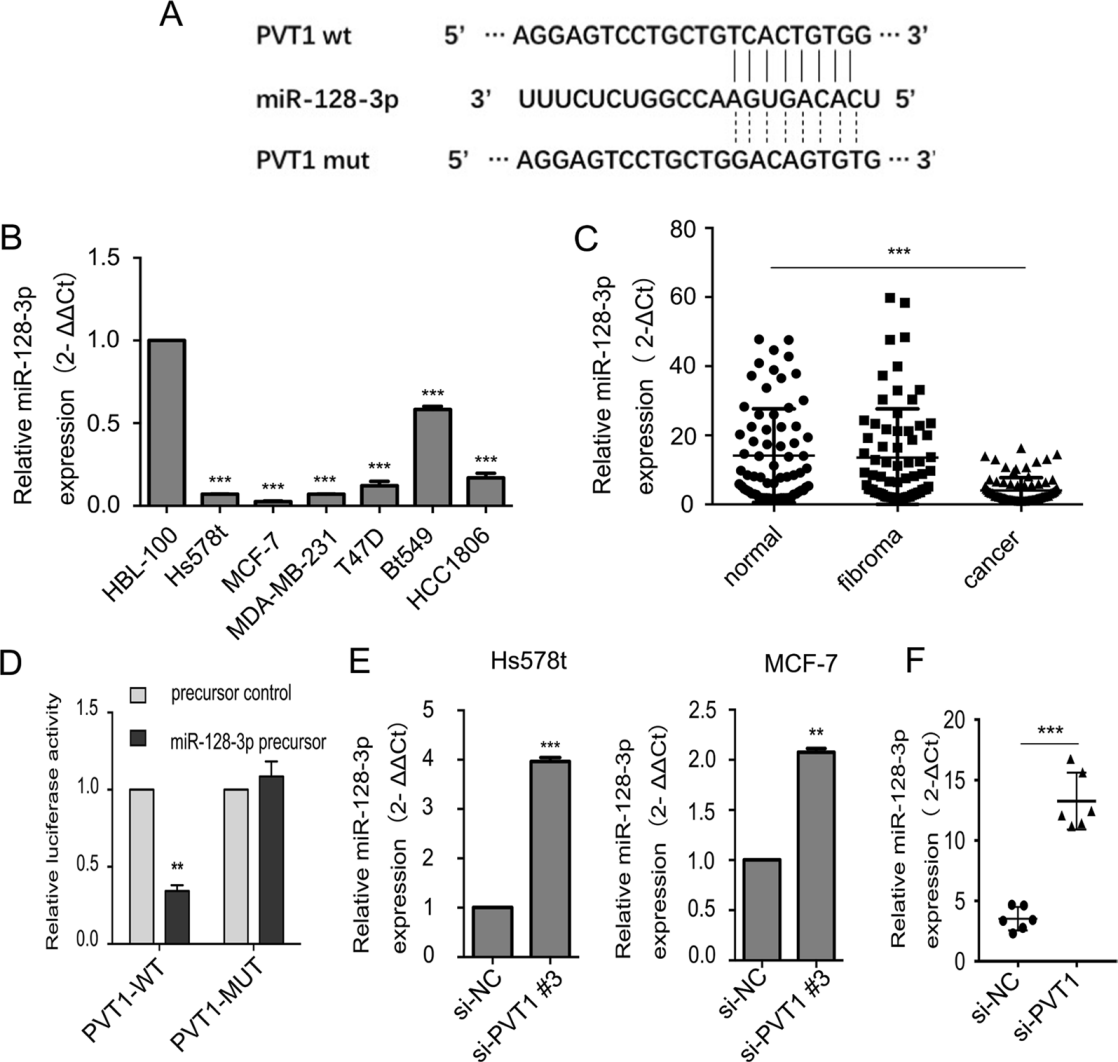
miR-128-3p was a target of PVT1 in BC cells. **A** The predicted interaction between PVT1 and miR-128-3p and the mutated miRNA binding site. **B** The expression levels of miR-128-3p in BC cell lines and the breast epithelial cell line HBL-100. **C** The plasma levels of miR-128-3p in 70 healthy controls, 70 fibroma patients, and 80 BC patients. **D** Luciferase reporter assays in 293 T cells. **E** The expression levels of miR-128-3p in Hs578t and MCF-7 cells with PVT1 knockdown. **F** The expression levels of miR-128-3p of tumor tissues from nude mice treated with si-NC and si-PVT1. Results were presented as the mean ± SD. **p < 0.01, ***P < 0.001

It is known that lncRNAs often function as competing endogenous RNAs (ceRNAs) or molecular sponges to recruit microRNAs (miRNAs), regulating their biological functions [13, 14]. To further explore the mechanism of PVT1 in BC tumorigenesis, we investigated whether miRNAs are involved. By using the online bioinformatics software -starBase (http://starbase.sysu.edu.cn), we observed that PVT1 contains a potential binding site for miR-128-3p (Fig. 4A). As shown in Fig. 4B, miR-128-3p expression in BC cell lines was lower than that in the immortalized mammary epithelial cell line HBL-100. The plasma level of miR-128-3p was much lower in BC patients than that in fibroma patients and healthy controls (Fig. 4C). We then performed a luciferase reporter assay, in which the Renilla luciferase (Rluc) was upstream of the PVT1 gene in one plasmid and the firefly luciferase as a control in another plasmid; the miR-128-3p binding site in Mut-PVT1 was disrupted. Both plasmids and a miR-128-3p expression plasmid were transfected into 293 T cells. As shown in Fig. 4D, the Rluc activity was significantly reduced with wild-type PVT1 and miR-128-3p. However, the Rluc activity with Mut-PVT1 and miR-128-3p showed no statistically significant changes. We next down-regulated PVT1 expression in Hs578t and MCF-7 cells using siRNAs and found PVT1 down-regulation markedly increased miR-128-3p levels (Fig. 4E). Similar results were also observed in xenografted tumors (Fig. 4F).

**Fig. 5:**
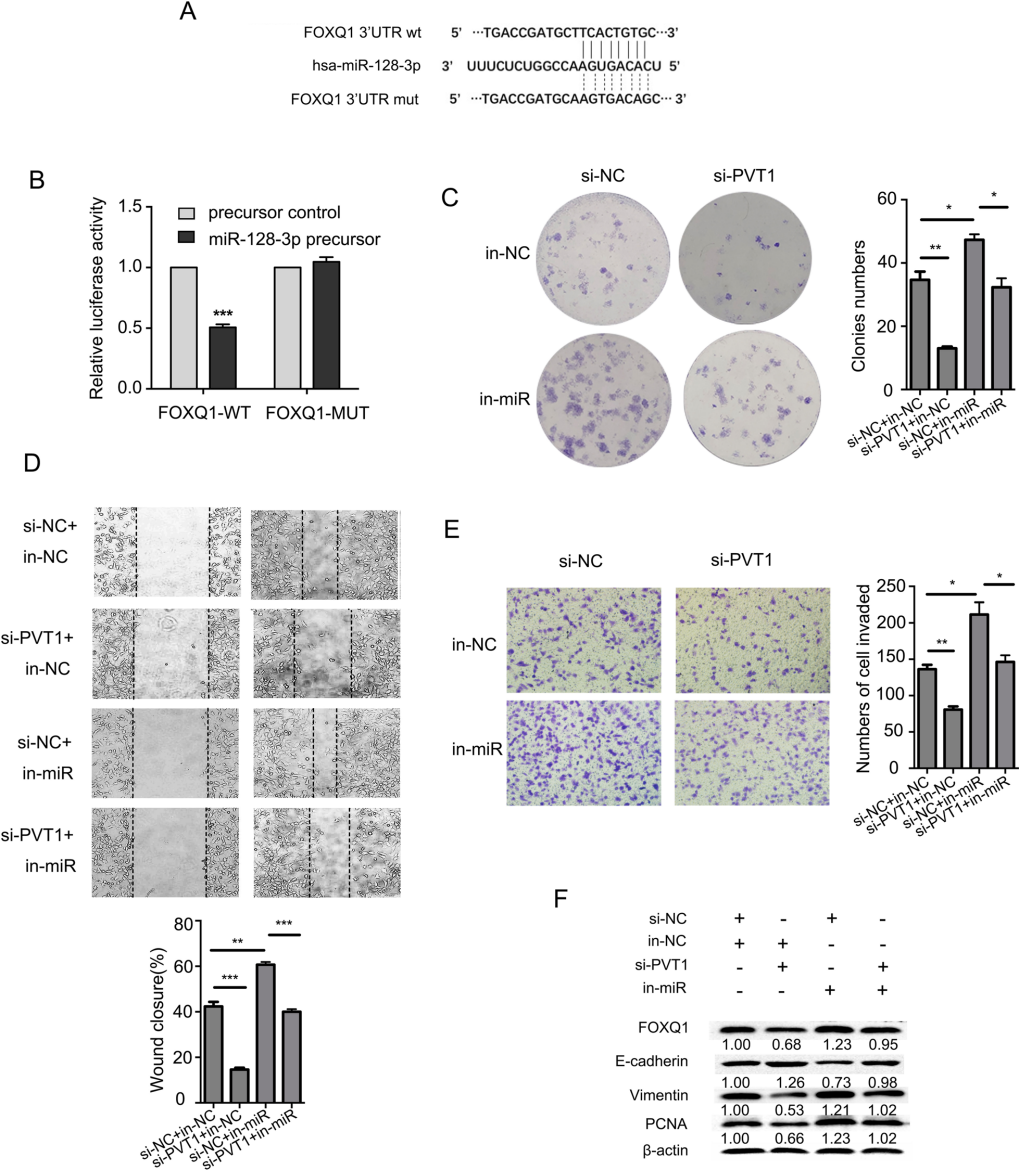
PVT1 acts as a sponge for miR-128-3p in BC cells. **A** The predicted interaction between miR-128-3p and FOXQ1 and the mutated binding site. **B** Luciferase reporter assays in 293 T cells. **C** Colony formation of Hs578t cells transfected with si-PVT1, miR-128-3p inhibitor, or both. **D** Wound healing assays for Hs578t cells. **E** Transwell assays for Hs578t cells. **F** Western blot assays for FOXQ1 and other proteins in Hs578t cells. Results were presented as the mean ± SD. *p < 0.05, **p < 0.01, ***P < 0.001

To identify target genes of miR-128-3p, we used starBase and TargetScan and found a potential miR-128-3p site in the FOXQ1 3΄UTR (Fig. 5A). FOXQ1 is a transcription factor involved in the EMT, which is associated with tumor growth and metastasis. A luciferase assay showed that miR-128-3p significantly inhibited reporter activity with the FOXQ1 3΄UTR; however, mutation in the putative targeting site in the 3΄UTR resulted in compete abrogation of the repressive effect (Fig. 5B). To examine whether PVT1 regulates FOXQ1 expression, BC cell proliferation and metastasis by directly targeting miR-128-3p, we transfected Hs578t cells with si-PVT1, miR-128-3p inhibitor, or both and evaluated the malignant phenotypes. As shown in Fig. 5C, the colony numbers of Hs578t cells were reduced with si-PVT1 transfected, but were increased with miR-128-3p knockdown. Yet with both miR-128-3p and PVT1 knockdown, the colony numbers were lower than that with miR-128-3p knockdown only. Moreover, knockdown of PVT1 markedly inhibited the wound closure rate and reduced cell invasion; inhibition of miR-128-3p exhibited the opposite effects, yet co-transfection with both inhibitors reversed increased migration and invasion of Hs578t cells mediated by miR-128-3p knockdown (Fig. 5D, E). When PVT1 was knocked down, the protein level of E-cadherin was elevated, whereas PCNA, Vimentin, and FOXQ1 were downregulated. Inhibition of miR-128-3p had opposite effects, and the impact of PVT1 knockdown was partially reversed by co-transfection with both inhibitors (Fig. 5F). These results suggest that PVT1 promotes BC cell proliferation, migration, and invasion, at least partially, by competitively binding to miR-128-3p.

### PVT1 promotes BC proliferation and metastasis by binding UPF1

**Fig. 6:**
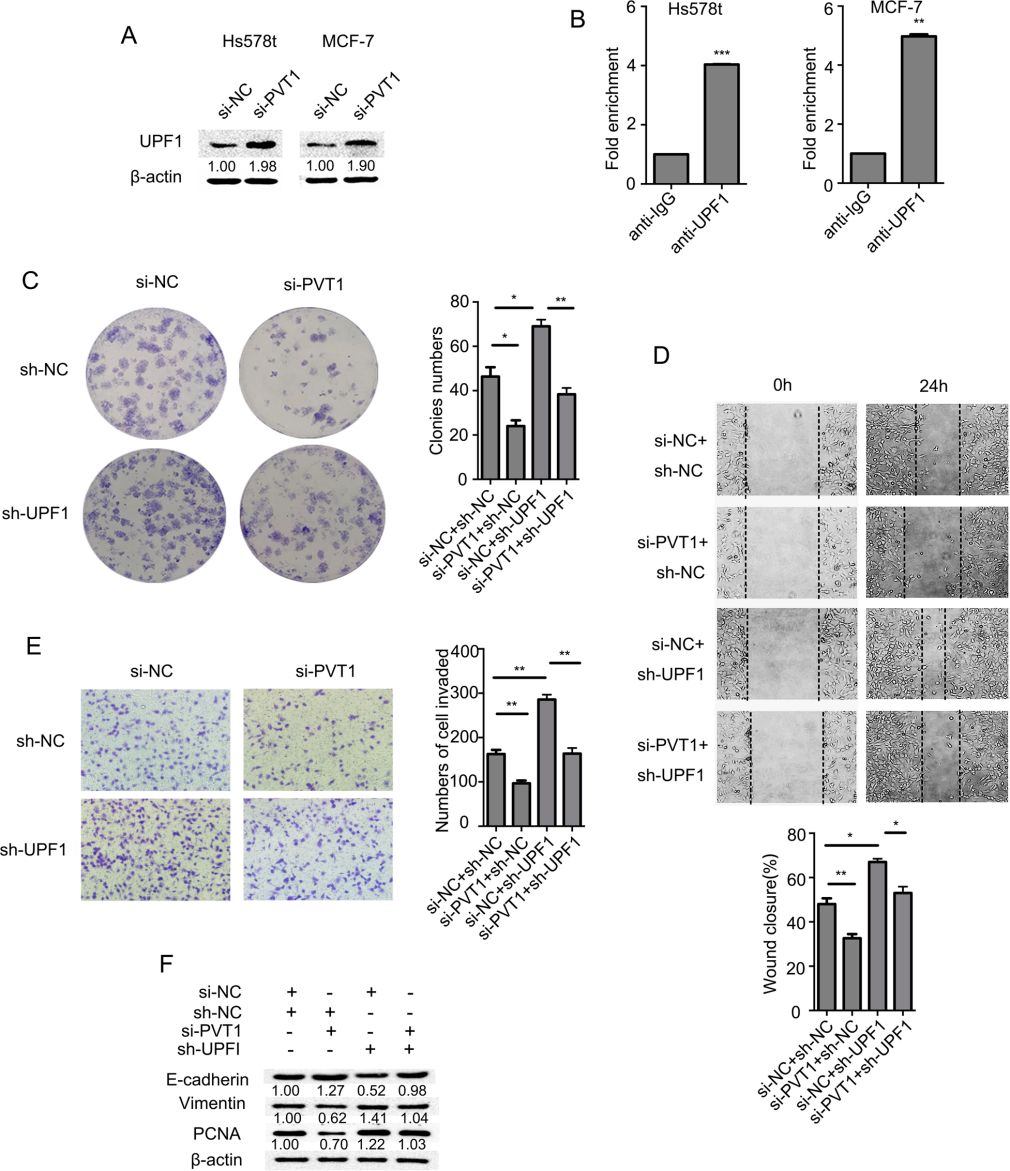
BC cell migration and proliferation are regulated by PVT1 and UPF1. **A** Western blot assays for UPF1 in Hs578t and MCF-7 cells with PVT1 knockdown. **B** RIP assays for UPF1 in Hs578t and MCF-7 cells. **C** Colony formation of Hs578t cells transfected with si-PVT1, sh-UPF1, or both. **D** Wound healing assays for Hs578t cells. **E** Transwell assays for Hs578t cells. **F** Western blot assays for proteins in Hs578t cells. Results were presented as the mean ± SD. *p < 0.05, **p < 0.01, ***P < 0.001

To further study the molecular mechanism underlying PVT1’s role in BC pathogenesis, we identified proteins potentially associated with PVT1 using starBase. Up-frameshift protein 1 (UPF1), a key factor in nonsense-mediated mRNA decay (NMD), is such a protein. UPF1 was upregulated when PVT1 was knocked down in Hs578t and MCF-7 cells (Fig. 6A). UPF1 antibody pulled down PVT1 in a RIP assay (Fig. 6B). To examine whether PVT1 regulates BC cell proliferation and metastasis by binding UPF1, we transfected Hs578t cells with si-PVT1#3, sh-UPF1#2 (sh-UPF1 #2 was the most efficient in knocking down UPF1 expression in Hs578t and MCF-7 cells, see Additional file 1: Figure B), or both, and evaluated the malignant phenotypes. As shown in Fig. 6C, PVT1 knockdown reduced colony formation of Hs578t cells, while UPF1 knockdown increased it. Yet inhibition of both UPF1 and PVT1 reversed the increase in colony formation mediated by UPF1 knockdown. Similar results were observed for migration and invasion (Fig. 6D, E). PVT1 knockdown upregulated E-cadherin, but down-regulated PCNA and vimentin. UPF1 knockdown had opposite effects, and the impact of PVT1 knockdown was partially reversed by concurrent UPF1 knockdown (Fig. 6F). These results suggest that PVT1 promotes BC cell proliferation, migration, and invasion by binding UPF1.

## Discussion

With the development of sequencing technology, many non-coding transcripts have been discovered. Among them, lncRNAs have attracted more and more attention given their wide range of functions. Increasing evidence indicates that abnormal expression of lncRNAs is critical for the development of cancers, including BC. PVT1 was reported to regulate cell proliferation and tumor growth in triple-negative BC through KLF5/ beta-catenin signaling [15]. In this study, we revealed that the oncogene lncRNA PVT1 plays a vital role in BC progression. We demonstrated that PVT1 was significantly upregulated in BC patients’ plasma, and its high levels in the circulation was correlated with poor prognosis. We further showed that PVT1 promoted BC cell proliferation, migration and invasion. Mechanistically, we found that PVT1 increased FOXQ1 expression via its sponge activity of miR-128-3p. In addition, PVT1 regulated UPF1 expression by directly binding it, providing added evidence to support PVT1 as an oncogene in BC.

Many lncRNAs function as ceRNAs or molecular sponges to recruit miRNAs, regulating their biological functions. Here we found that PVT1 upregulated FOXQ1 by competitively binding to miR-128-3p, thereby promoting BC cell proliferation and migration. miR-128-3p was upregulated by PVT1 knockdown, which also lead to repression of FOXQ1, a target gene of miR-128-3p. Therefore, the effect of PVT1 on BC cell proliferation and migration could be explained in part by its function as a molecular sponge for miR-128-3p. miR-128-3p exhibits a tumor suppressor role in human malignancies [16]. We found that miR-128-3p was significantly downregulated in BC. FOXQ1, a member of the human Forkhead-Box (Fox) gene family that consists of at least 43 members, induces EMT though repressing E-cadherin expression by targeting the E-box in its promoter region [17]. Higher expression of FOXQ1 was found in multiple cancers, including BC, suggesting that FOXQ1 may play an essential role associated with EMT [18]. Our findings suggest that PVT1 may promote BC tumorigenesis by acting as a ceRNA that competitively binds to miR-128-3p and upregulates FOXQ1 expression.

lncRNAs often to specific protein partners directly to influence their activity or localization. UPF1 is an RNA-dependent helicase and ATPase that is required for NMD of mRNAs containing premature stop codons and takes part in cancer progression. Liu and colleagues found that UPF1 gene is commonly mutated in Pancreatic adenosquamous carcinoma [19]. Previous studies showed that UPF1 inhibits the hepatocellular carcinoma progression by targeting MRP2/ABCC2 or long non-coding RNA UCA1 [20, 21]. Cao and colleagues found that UPF1 may inhibit TGF-β signaling by decreased expression of Smad2/3, MIXL1 and SOX17 in lung adenocarcinoma [22]. Increasing evidence has shown that UPF1 is significantly downregulated in several cancers, showing that NMD is attenuated to permit oncogenesis; UPF1 downregulation promotes EMT in cancer cells [20, 22]. In our study, we revealed that PVT1 directly bound to UPF1 and inhibited its expression, thereby promoting BC cell proliferation, migration and invasion.

**Fig. 7:**
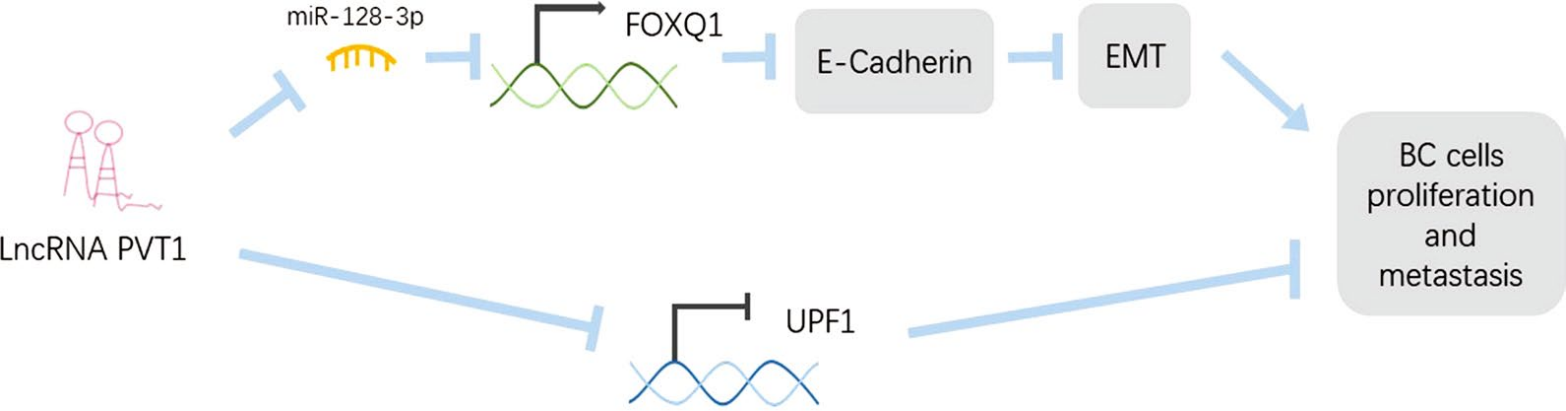
Schematic model of PVT1 promotes tumor cell proliferation and metastasis in BC

In summary, our findings support that PVT1 acts as an oncogene in BC through binding miR-128-3p and UPF1 and that PVT1 is a potential target for BC therapeutic development (Fig. 7).

## Conclusion

Taken together, the result of this study indicates that PVT1 functions as an oncogene in breast cancer. High PVT1 expression is associated with tumor progression and poor prognosis. PVT1 promotes breast cancer proliferation and metastasis. As direct targets of PVT1, UPF1 and miR-128-3p mediate the roles of PVT1 in tumor proliferation and metastasis. The effect of PVT1 on breast cancer progression suggests that PVT1 has potential use in antitumor therapies and deserves further investigation.

## Supplementary Information

**Additional file 1:** table: Association between PVT1 expression and the subtypes of breast cancer patients (all female). Figure: si-PVT1 and sh-UPF1 knockdown efficiency in Hs578t and MCF-7 cells. (A) Western blot assays for FOXQ1 and UPF1 in Hs578t and MCF-7 cells. (B) Western blot assays for UPF1 in Hs578t and MCF-7 cells.

## Data Availability

All data generated or analyzed during this study are included in this published article.

## Abbreviations

BC: Breast cancer
NPC: Nasopharyngeal carcinoma
PVT1: Plasmacytoma variant translocation 1
UPF1: Up-frameshift protein 1
FOXQ1: Forkhead box Q1
lncRNAs: Long noncoding RNAs
miRNAs: MicroRNAs
ceRNAs: Competitive endogenous RNAs
EMT: Epithelial–mesenchymal transition
siRNA: Small interfering RNA
shRNA: Small hairpin RNA
EDTA: Ethylene diamine tetraacetic acid
PBS: Phosphate-buffered saline
UTR: Untranslated region
qRT-PCR: Quantitative real-time polymerase chain reaction
RIP: RNA immunoprecipitation
IHC: Immunohistochemistry
