# Correction: Long noncoding RNA PVT1 promotes breast cancer proliferation and metastasis by binding miR-128-3p and UPF1

**DOI:** 10.1186/s13058-023-01698-1

**Published:** 2023-08-21

**Authors:** Shuiyi Liu, Weiqun Chen, Hui Hu, Tianzhu Zhang, Tangwei Wu, Xiaoyi Li, Yong Li, Qinzhi Kong, Hongda Lu, Zhongxin Lu

**Affiliations:** 1 Department of Medical Laboratory, The Central Hospital of Wuhan, Tongji Medical College, Huazhong University of Science and Technology, 26 Shengli St., Jiangan District, Wuhan, 430014 China; 2 Cancer Research Institute of Wuhan, The Central Hospital of Wuhan, Tongji Medical College, Huazhong University of Science and Technology, Wuhan, 430014 China; 3 Key Laboratory for Molecular Diagnosis of Hubei Province, The Central Hospital of Wuhan, Tongji Medical College, Huazhong University of Science and Technology, Wuhan, 430014 China; 4 School of Laboratory Medicine, Hubei University of Chinese Medicine, Wuhan, 430065 China; 5 Department of Medicine, Dan L Duncan Comprehensive Cancer Center, Baylor College of Medicine, Houston, TX 77030 USA; 6 Department of Oncology, The Central Hospital of Wuhan, Tongji Medical College, Huazhong University of Science and Technology, Wuhan, 430014 China

**Correction: Breast Cancer Research (2021) 23:115** 10.1186/s13058-021-01491-y

The original publication of this article contained an incorrect affiliation for author Yong Li. The incorrect and correct information is shown in this correction article, the original article has been updated.

Incorrect

Department of Medical Laboratory, The Central Hospital of Wuhan, Tongji Medical College, Huazhong University of Science and Technology, 26 Shengli St., Jiangan District, Wuhan, 430014, China

Department of Medicine, Dan L Duncan Comprehensive Cancer Center, Baylor College of Medicine, Houston, TX, 77030, USA

Correct

Department of Medicine, Dan L Duncan Comprehensive Cancer Center, Baylor College of Medicine, Houston, TX, 77030, USA

