# Supplementary material for: Long noncoding RNA PVT1 promotes breast cancer proliferation and metastasis by binding miR-128-3p and UPF1
Source: Breast Cancer Res. 2021 Dec 18;23:115. doi: 10.1186/s13058-021-01491-y (PMC8684126; doi:10.1186/s13058-021-01491-y)
Supplement: Supplementary file 1 — Additional file 1: table: Association between PVT1 expression and the subtypes of breast cancer patients (all female). Figure: si-PVT1 and sh-UPF1 knockdown efficiency in Hs578t and MCF-7 cells. (A) Western blot assays for FOXQ1 and UPF1 in Hs578t and MCF-7 cells. (B) Western blot assays for UPF1 in Hs578t and MCF-7 cells. [file 13058_2021_1491_MOESM1_ESM.pdf]

# Additional file 1

## Additional table:

Additional table: Association between PVT1 expression and the subtypes of breast cancer patients (all female)

| Characteristics | Case（n=80） | Expression of PVT1 | | P value |
|---|---|---|---|---|
| | | Low (n=40) | High (n=40) | |
| subtype | | | | |
| Luminal A | 29 | 17 | 12 | 1.000 |
| Luminal B | 19 | 8 | 11 | |
| Her-2+ | 17 | 8 | 9 | |
| Basal-like | 15 | 7 | 8 | |

## Additional figure:

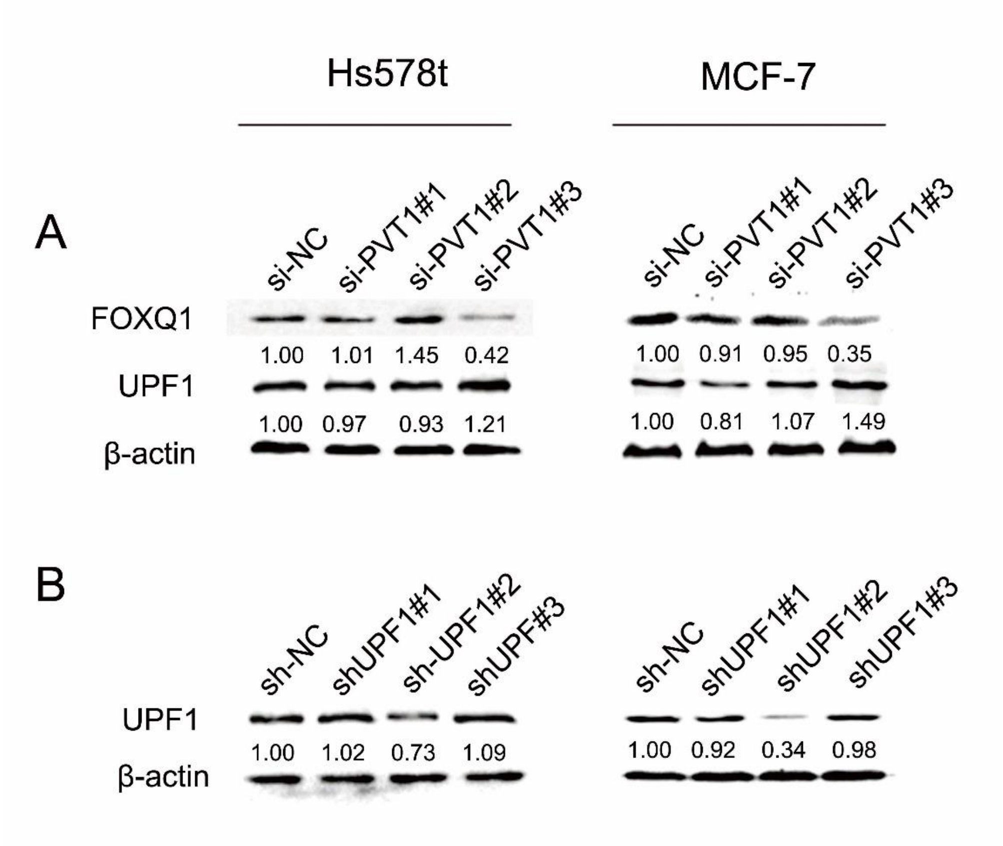


**Additional figure: si-PVT1 and sh-UPF1 knockdown efficiency in Hs578t and MCF-7 cells**

**(A)** Western blot assays for FOXQ1 and UPF1 in Hs578t and MCF-7 cells. **(B)** Western blot assays for UPF1 in Hs578t and MCF-7 cells.
